# Thirty years of population-based breast cancer screening in Iceland: a comparison of quality indicators and tumour characteristics between women aged 40–49 and 50–69 years

**DOI:** 10.2340/1651-226X.2025.44090

**Published:** 2025-08-14

**Authors:** Alfheidur Haraldsdottir, Helgi Birgisson, Agust I. Agustsson, Laufey Tryggvadottir

**Affiliations:** a Icelandic Cancer Registry, Icelandic Cancer Society, Reykjavik, Iceland; b Cancer Screening Coordination Centre, Primary Health Care of the Capital Area, Reykjavik, Iceland; c Faculty of Medicine, University of Iceland, Reykjavik, Iceland

**Keywords:** Breast cancer, breast cancer screening, early detection of cancer, quality indicators

## Abstract

**Background and purpose:**

Organised mammography screening reduces breast cancer mortality by 30–40% in women aged 50–69. Despite limited evidence for women aged 40–49, screening guidelines are trending toward younger ages. Iceland has offered biennial screening to women aged 40–69 since 1987. This study compares screening quality indicators and tumour characteristics between women aged 40–49 and 50–69 from 1990 to 2020.

**Patient/material and methods:**

Screening-related data were obtained from the Icelandic Breast Cancer Screening Program, and breast cancer diagnoses and tumour characteristics were sourced from the Icelandic Cancer Registry.

**Results:**

In total, 84,677 women aged 40–69 years attended 455,532 organised screening sessions in Iceland over a 30-year period. Women aged 40–49 years demonstrated higher recall rates (4.9% vs. 3.5%) and lower participation rates (60.7% vs. 61.5%), lower breast cancer detection rates (2.1 vs. 6.0/1,000), and lower episode sensitivity (54.8% vs. 70.5%), compared to those aged 50–69 years. Among screen-detected cases, women aged 40–49 years exhibited a higher proportion of tumours larger than 20 mm (29.7% vs. 21.7%), more lymph node positivity (41.2% vs. 28.2%) and higher human epidermal growth factor receptor 2 (HER2) positivity (18.6% vs. 11.8%), compared to those aged 50–69 years.

**Interpretation:**

The disparity in breast cancer screening performances between the age groups may reflect unmodifiable factors in younger women. The presence of advanced tumour characteristics among women aged 40–49 years who attended screening indicates the importance of early detection for improving prognosis.

## Thirty years of population-based breast cancer screening in Iceland: a comparison of quality indicators and tumour characteristics between women aged 40–49 and 50–69 years

**Figure:**
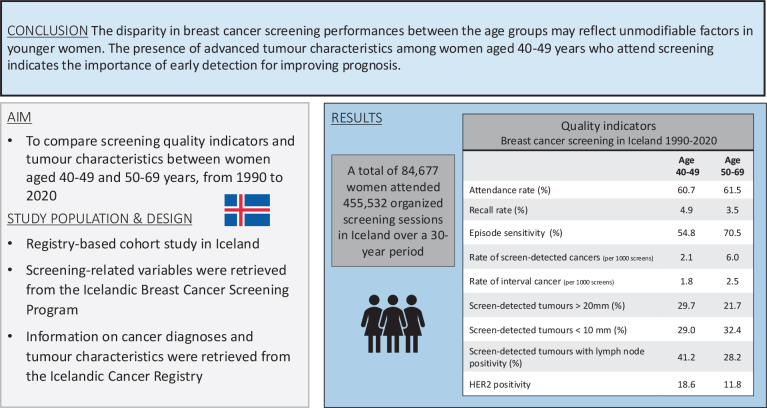

## Introduction

Organised breast cancer screening is a widely used strategy that aims to reduce mortality and morbidity from breast cancer through early detection. Screening is estimated to reduce breast cancer mortality by 30–40% in women aged 50–69 years [1], which has been the recommended target age from the onset of breast cancer screening. Substantial risk reduction has also been reported in women aged 70–74 years [2, 3]. The European Commission Initiative on Breast Cancer (ECIBC) has recommended expanding the screening range, lowering the starting age from 50 to 45 years and raising the upper age limit from 69 to 74 years [4]. The U.S. Preventive Services Task Force (USPSTF) has lowered the recommended starting age to 40 years [5]. This is subject to debate [6] as evidence for mortality reduction is limited for women aged 40–49 years [1] and there is uncertainty regarding the proportion of overdiagnosis for this age group [7, 8]. However, women aged 40–49 often present with more advanced disease at diagnosis, compared with women who are diagnosed at age 50 years or older [9–11].

To achieve the intended outcomes of screening, it is essential to meet defined quality standards and use standardised quality indicators to maintain an appropriate balance between its benefits and potential harms [12, 13]. Studies on quality of breast cancer screening programs for women aged 40–49 years are scarce, as organised population-based screening for this age group is uncommon. In Iceland, an organised nationwide population-based breast cancer screening program for women aged 40–69 years has been in operation since 1987.

Given the long-standing inclusion of women aged 40–69 years in the Icelandic National Breast Cancer Screening Program, an approach that was uncommon during much of the study period and continues to be questioned, the objective of this study was to compare selected key quality indicators for breast cancer screening between women aged 40–49 and 50–69 years during the years 1990–2020. A secondary aim was to compare certain tumour characteristics among screen-detected, interval, and clinically diagnosed breast cancers across these two age groups.

## Patients/material and methods

This study is a registry-based observational cohort study where information on screening-related variables was retrieved from the Icelandic Breast Cancer Screening Program, an organised nationwide screening program run by the Icelandic Cancer Society (ICS) from 1987–2020. The Greater Reykjavik area was covered by facilities located at the premises of the ICS, the northern part of the country was covered by Akureyri Hospital and mobile screening units served women living in rural areas of the country. The ICS kept a centralised database registering all mammographies performed in Iceland during the years 1987–2020. From the onset, the target population comprised women aged 40–69 years residing in Iceland who received biennial invitation letters. As a result, approximately half of the target population was invited each year. For this study we used data from all women in this age range who ever attended the screening program between January 1^st^ 1990 and December 31^st^ 2020, corresponding to women born between 1919 and 1980. We also included women who were clinically diagnosed, defined as women who had not attended breast cancer screening within the 24 months prior to diagnosis. Information on breast cancer diagnoses between 1990 and 2020 was ascertained from the nationwide Icelandic Cancer Registry, along with information on oestrogen receptor status (ER), HER2 status, lymph node involvement and tumour size.

## Statistical analysis

The quality indicators analysed in this study were participation rate, recall rate, breast cancer detection rate, interval cancer rate, positive predictive value (PPV-1), tumour size, lymph node status, and episode sensitivity.

The screening attendance rate was calculated as the number of women who attended screening, divided by the estimated number of women who were considered eligible for screening. Eligibility was defined as the number of women aged 40–69 years, who were invited biennially for screening. The estimated number of women eligible for screening was derived from population data provided by Statistics Iceland [14]. A recall for further assessment due to abnormal mammography was defined as screening-related if it occurred within 180 days after screening attendance. The recall rate was the proportion of the women who were called back for further assessment of those who participated in the screening. A false-positive recall was defined as a recall that was not followed by breast cancer diagnosis.

Screen-detected breast cancers were defined as ductal carcinoma in situ (DCIS) or invasive breast cancer diagnosed within 180 days (6 months) after screening mammography [15, 16] or 30 days before the estimated next screening date [17]. However, if the screening mammogram was negative and/or clinical attendance was documented prior to the date of diagnosis, the cancer was considered as interval cancer, despite being diagnosed within 180 days after screening mammography. Interval cancers were also defined as either DCIS or invasive cancers diagnosed between 6 and 23 months after screening attendance. The annual breast cancer detection rate was calculated by dividing the number of screen-detected cancers by the total number of women screened that same year and presented as a rate per 1,000 screened women. The interval cancer rate was calculated by dividing the number of interval cancers per year by the number of women with a negative screening result in the prior year. Clinically diagnosed breast cancers were defined as such if DCIS or invasive breast cancer was identified in women who had not participated in breast cancer screening within the previous 24 months.

PPV-1 was calculated as the proportion of women who were diagnosed with breast cancer after being recalled after screening. Episode sensitivity, a metric that reflects the ability of the screening program to detect cancer within the screening interval [12], was defined as the proportion of screen-detected breast cancer divided by the sum of screen-detected cancer and interval cancer.

The attendance rate, recall rate, breast cancer detection rate, interval cancer rate and episode sensitivity were presented as 2-year running averages throughout the study period. Screening attendance was also analysed on a yearly basis. Rates for attendance, recall, breast cancer detection, interval cancer, and PPV-1 were shown as combined total, prevalent and subsequent rate/percentages. Prevalent was defined as first attendance and subsequent refers to all later attendances. Invasive screen-detected cancers, interval cancers and clinically diagnosed breast cancers were further subcategorised by ER status (positive/negative), HER2 receptor status (positive/negative), tumours size (smaller than 10 mm/10–20 mm/larger than 20 mm) and lymph node status (positive/negative). Tumour size and lymph node involvement for screen-detected cancers were also shown as prevalent and subsequent percentages.

The age groups analysed were women between 40–49 years and 50–69 years. The age group 40–49 years was further divided into two subgroups of women aged 40–44 years and 45–49 years. We used the age group 50–69 as a reference group for comparisons. We also performed subgroup comparisons between women aged 40–44 and 45–49 years.

European guidelines for quality assurance in breast cancer screening and diagnosis were used as reference where applicable [17]. Chi-square test was used for comparing proportions. All statistical tests were two-sided and *p*-values less than 0.05 were considered statistically significant.

This study is reported according to the Strengthening the Reporting of Observational Studies in Epidemiology (STROBE) guideline. R-studio and Microsoft Excel were used for statistical analyses. The study was approved by the National Bioethics Committee (VSNb2020110001/03.0l).

## Results

**Figure 1:**
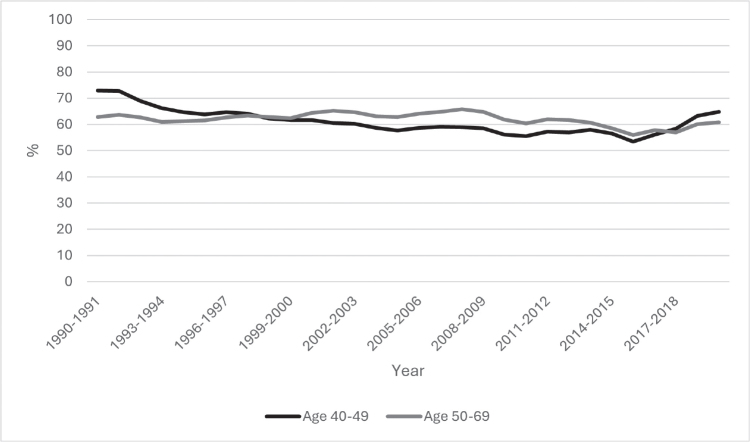
The breast cancer screening attendance rate for Icelandic women aged 40–49 and 50–69 years during the years 1990–2020 (shown as 2-year running averages).

Over a 30-year period, 84,677 women aged 40–69 years attended 455,532 organised screening sessions. On average, each woman participated in 4.7 screening rounds. The attendance rate remained stable throughout the study period, although overall attendance among women aged 40–49 years was slightly lower at 60.7% while it was 61.5% among women aged 50–69 years (see Table 1 and Figure 1). When looking at single years instead of the running 2-year averages, attendance increased significantly between the years 2018 and 2019 for both age groups, or 53.2% versus 65.2% for women aged 50–69 years and 55.6% versus 67.9% for women aged 40–49 years (see Supplementary Figure 1). Of all the mammograms performed during the study period in the age group 50–69 years, 4.5% were prevalent screens. This proportion was 29.8% for the age group 40–49 years.

**Table 1:** Population-based breast cancer screening in Iceland during 1990–2020 – quality indicators among women aged 50–69 and 40–49 years old.

Quality indicators	Age 50–69	Age 40–49	*p*	Subgroups of age 40–49				
				Age 45–49	*p*	Age 40–44	*p*	Acceptable/Desirable level^µ^
Attendance, no, %	271,836 (61.5)	183,696 (60.7)	< 0.001	94,792 (60.2)	< 0.001	88,904 (60.4)	< 0.001	70/75
- Prevalent screen, no (%)	12,305 (4.5)	54,757 (29.8)	< 0.001	46,175 (49.3)	< 0.001	8,042 (9.0)	< 0.001	
- Subsequent screens	25,9531 (95.5)	128,939 (70.2)	< 0.001	48,077 (50.7)	< 0.001	80,862 (91.0)	< 0.001	
Age at screening, mean (SD)	58.5 (5.6)	44.4 (2.9)	< 0.001	47,0 (1.4)	< 0.001	41.9 (1.5)	< 0.001	
Recall rate, no. (%)								
- All screens	9,633 (3.5)	8,993 (4.9)	< 0.001	4,222 (4.7)	< 0.001	4,771 (5.0)	< 0.001	
- Prevalent screen	677 (5.5)	3,515 (6.4)	< 0.001	640 (8.0)	< 0.001	2,875 (6.2)	0.007	< 7/5
- Subsequent screens	8,956 (3.5)	5,478 (4.2)	< 0.001	3,582 (4.4)	< 0.001	1,896 (3.9)	< 0.001	< 5/3
PPV-1, no. (%)								
- All screens	1,580 (16.4)	375 (4.2)	< 0.001	237 (5.6)	< 0.001	138 (2.9)	< 0.001	
- Prevalent screen	96 (14.2)	123 (3.5)	< 0.001	47 (7.3)	< 0.001	76 (2.6)	< 0.001	
- Subsequent screens	1,484 (16.6)	252 (4.6)	< 0.001	190 (5.3)	< 0.001	62 (3.3)	< 0.001	
Rate of screen-detected cancer, per 1,000								
- All screens	6.0	2.1	< 0.001	2.8	< 0.001	1.5	< 0.001	
- Prevalent screen	8.5	2.4	< 0.001	6.1	0.057	1.7	< 0.001	
- Subsequent screens	5.9	2.1	< 0.001	2.5	< 0.001	1.4	< 0.001	
Rate of interval cancer, per 1,000								
- All screens	2.5	1.8	< 0.001	2.0	0.003	1.6	< 0.001	
- Prevalent screen	2.1	1.7	< 0.001	2.4	0.717	1.6	0.175	
- Subsequent screens	2.5	1.8	< 0.001	1.9	0.001	1.6	< 0.001	

Age-group 50–69 used as a reference group.

PPV-1: Positive predictive value.

^µ^Acceptable/desirable level according to European guidelines for quality assurance in breast cancer screening – Fourth edition [18].

**Figure 2:**
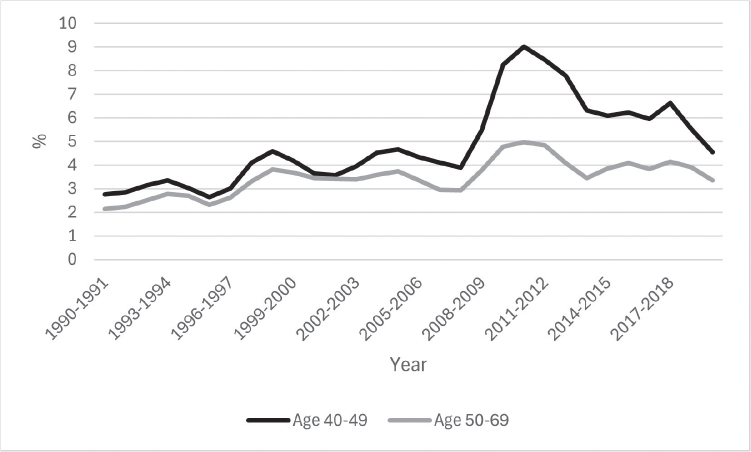
Recalls after organised nationwide breast cancer screening for Icelandic women aged 40–49 and 50–69 years, during the period 1990–2020.

During the study period, the overall recall rate was higher among women aged 40–49 years at 4.9% compared to 3.5% in the 50–69 age group, particularly during the years 2010–2015 (see Figure 2). However, women aged 50–69 years had higher PPV-1 or 16.4%, while this proportion was only 4.2% for women aged 40–49 years.

**Figure 3:**
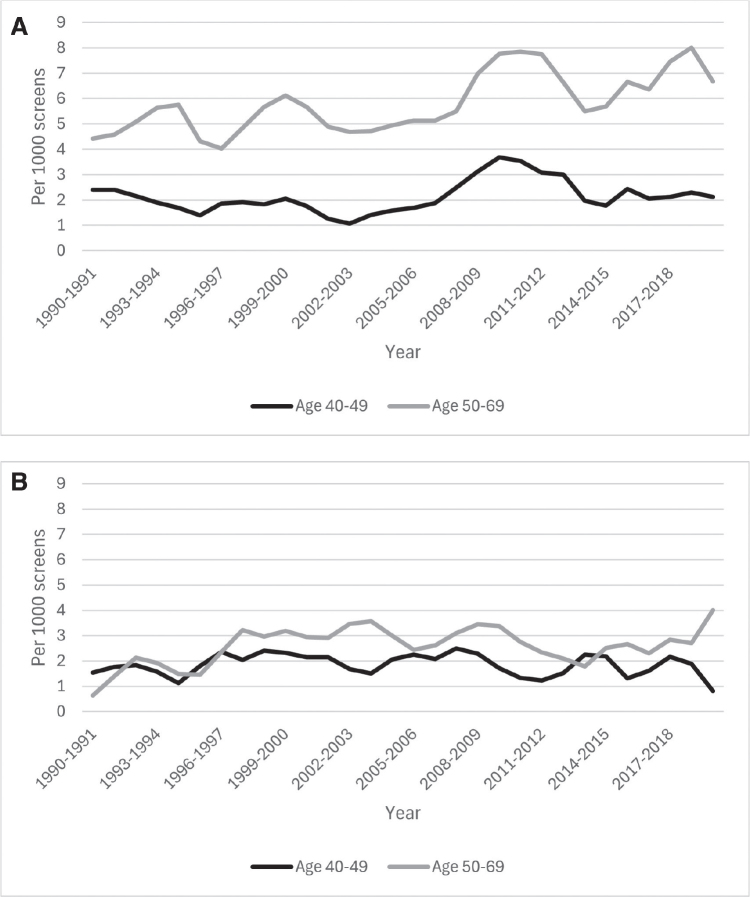
The rate of breast cancer detection (A) and interval cancer (B) for women aged 50–69 years and 40–49 years during the period 1990–2020.

The breast cancer detection rate was overall lower among women aged 40–49 years, or 2.1 per 1,000 screens, while this rate was 6.0 per 1,000 screens among women aged 50–69 years. However, the interval cancer rate was also higher among women aged 50–69 years, or 2.5 per 1,000 screens against 1.8 per 1,000 for women aged 40–49 years. Figures 3A and B show rates for screen-detected and interval cancer during the study period. Except for a notable increase in detection rate during the years 2010–2015, both breast cancer detection and interval cancer rates remained relatively stable for both age groups. However, the proportion of interval cancer was higher among younger women, at 45.2% versus 29.5% among older women during the study period. We observed very similar trends in parameters between the age groups for prevalent and subsequent screens (see Table 1).

When the subgroups of women aged 45–49 and 40–44 years, were compared with women aged 50–69 years, similar trends were observed in both subgroups as seen in the overall 40-49 age group. The only exception was that no difference was observed for the detection rate of prevalent breast cancers between women aged 50–69 and those aged 45–49 years. When women aged 40–44 years and 45–49 years were compared, the younger group had significantly lower proportion of PPV-1 and lower breast cancer detection rate for both prevalent and subsequent screens. No significant difference was observed for interval cancer rate between the subgroups (Supplementary Table 1B).

Table 2 shows tumour characteristics among women aged 50–69 and 40–49 years with screen-detected, interval and clinically diagnosed breast cancer. For screen-detected cancers, women aged 40–49 years had higher proportion of in situ tumours (19.2%), tumours over 20 mm (29.7%), positive lymph nodes (41.2%) and tumours with positive HER2 receptors, when compared with older women. This difference remained significant for larger tumours and lymph node involvement in subsequent screening rounds. Among interval cancers, the only observed difference between age groups was in lymph node positivity, where women aged 40–49 had a higher proportion, or 54.3% compared with 41.8%. Apart from a slight difference in tumour diameter, no significant differences were observed between the age groups among clinically detected cancers.

**Table 2:** Tumour characteristics among women with screen-detected, interval and clinically diagnosed invasive breast cancer 1990–2020.

Tumour characteristics	Screen-detected breast cancers (*n* = 2,018)							Acceptable/ desirable level^µ^	Interval breast cancers (*n* = 1,006)							Clinically diagnosed breast cancer (*n* = 1,236)						
	Age	Age	*p*	Subgroups of age 40–49					Age	Age	*p*	Subgroups of age 40–49				Age	Age	*p*	Subgroups of age 40–49			
	50–69	40–49		Age 45–49	*p*	Age 40–44	*p*	50–69	40–49	Age 45–49		*p*	Age 40–44	*p*	50–69	40–49	Age 45–49		*p*	Age 40–44	*p*
**All tumours, no**	1,624	394		248		146			681	325		173		152		878	358		192		166	
**Type (%)**																						
In situ	212 (13.1)	76 (19.2)	0.002	47 (19.0)	0.012	29 (19.9)	0.022		59 (8.7)	28 (8.6)	0.980	11 (6.4)	0.392	17 (11.2)	0.324	32 (3.6)	15 (4.2)	0.649	5 (2.6)	0.475	10 (6.0)	0.153
Invasive*	1,412 (86.9)	318 (80.7)		201 (81.0)		117 (80.1)		90/80–90	622 (91.3)	297 (91.4)		162 (93.6)		135 (88.8)		846 (96.4)	343 (95.8)		187 (97.4)		156 (94.0)	
**Tumour diameter, mm**																						
Median	14.0	15.0	0.004	15.0	0.032	15.5	0.031		18.0	19.0	0.444	18.0	0.754	20.0	0.363	22.0	21.0	< 0.001	20.0	< 0.001	21.0	< 0.001
Mean	16.1	18.5	0.002	18.2	0.022	19.1	0.027		22.5	22.8	0.787	23.1	0.740	22.5	0.960	26.9	27.2	< 0.001	27.9	< 0.001	26.3	< 0.001
***All* <10 (%)**	447 (32.4)	90 (29.0)	0.254	56 (28.0)	0.249	34 (30.4)	0.661		107 (18.3)	46 (16.3)	0.467	27 (17.3)	0.770	19 (15.1)	0.388	96 (12.6)	46 (14.6)	0.509	26 (15.0)	0.729	19 (13.4)	0.489
*Prevalent screen*	27 (31.8)	25 (25.5)	0.350	9 (24.3)	0.408	16 (26.2)	0.469	Na/ ≥ 25														
*Subsequent screens*	420 (32.4)	65 (30.6)	0.603	47 (29.1)	0.410	18 (35.3)	0.666	≥ 25/≥ 30														
***All* 10 ≥, < 21, *n* (%)**	634 (45.9)	128 (41.3)	0.140	86 (43.4)	0.513	42 (37.5)	0.086		245 (41.9)	113 (40.1)	0.612	64 (41.0)	0.848	49 (38.9)	0.536	258 (34.0)	113 (35.9)	0.612	63 (36.4)	0.545	50 (35.2)	0.779
*Prevalent screen*	27 (31.8)	39 (39.7)	0.259	15 (40.5)	0.348	24 (39.3)	0.343															
*Subsequent screens*	607 (46.8)	89 (41.9)	0.189	71 (44.1)	0.511	18 (35.3)	0.105															
***All* >20, *n* (%)**	300 (21.7)	92 (29.7)	0.003	56 (28.3)	0.039	36 (32.1)	0.011		233 (39.8)	123 (43.6)	0.275	65 (41.7)	0.661	58 (46.0)	0.192	405 (53.4)	157 (49.8)	0.293	84 (48.6)	0.254	73 (51.4)	0.669
*Prevalent screen*	31 (36.5)	34 (34.6)	0.802	13 (35.1)	0.888	21 (34.4)	0.799															
*Subsequent screens*	269 (20.8)	58 (27.3)	0.031	43 (26.7)	0.0825	15 (29.4)	0.137															
NA, *n*	31	8	0.728	3	0.516	5	0.154		37	15	0.582	6	0.264	9	0.752	87	28	0.262	14	0.244	14	0.618
**Positive node status, *n* (%)**																						
***All***	385 (28.2)	127 (41.2)	< 0.001	77 (39.1)	0.002	50 (45.0)	< 0.001		239 (41.8)	152 (54.3)	< 0.001	84 (53.8)	0.007	68 (54.8)	0.008	377 (50.8)	143 (46.1)	0.166	81 (46.8)	0.432	62 (45.3)	0.232
*Prevalent screen*	39 (46.4)	45 (45.9)	0.814	16 (43.2)	0.923	29 (47.5)	0.687	NA /< 30														
**ER, *n* (%)**																						
Positive	1,115 (84.9)	250 (83.3)	0.510	165 (85.5)	0.817	85 (79.4)	0.137		419 (71.1)	200 (70.9)	0.948	113 (72.9)	0.664	87 (68.5)	0.554	585 (76.6)	229 (72.0)	0.114	126 (71.6)	0.176	103 (72.5)	0.302
NA, *n*	98	18	0.410	8	0.113	10	0.515		33	15	0.871	7	0.612	8	0.772	82	25		11		14	0.779
HER2, *n* (%) **																						
Positive	114 (11.8)	38 (18.6)	0.008	8 (22.6)	<0.001	8 (11.3)	0.893		79 (20.1)	28 (16.8)	0.365	20 (21.3)	0.791	8 (11.0)	0.073	88 (17.3)	33 (6.5)	0.983	19 (3.7)	0.099	14 (2.7)	0.859
NA, *n*	76	16	0.991	9	0.679	7	0.585		51	15	0.233	8		7	0.476	72	29	0.774	14	0.807	15	0.467

Age group 50–69 years used as a reference group. Values that are not available are not included when calculating the percentage of distributions.

* Information on tumour diameter, lymph node status, oestrogen receptors status (ER), HER2 status is for invasive tumours only.

** Assessment of HER2 status started in 2003.

µ Acceptable/desirable level according to European guidelines for quality assurance in breast cancer screening – Fourth edition [18].

With the exception of a small difference in lymph node status for subsequent tumours, no differences were observed in tumour characteristics for screen-detected, interval, or clinically diagnosed cancers between women aged 40–44 and those aged 45–49 years (Supplementary Table 1B).

**Figure 4:**
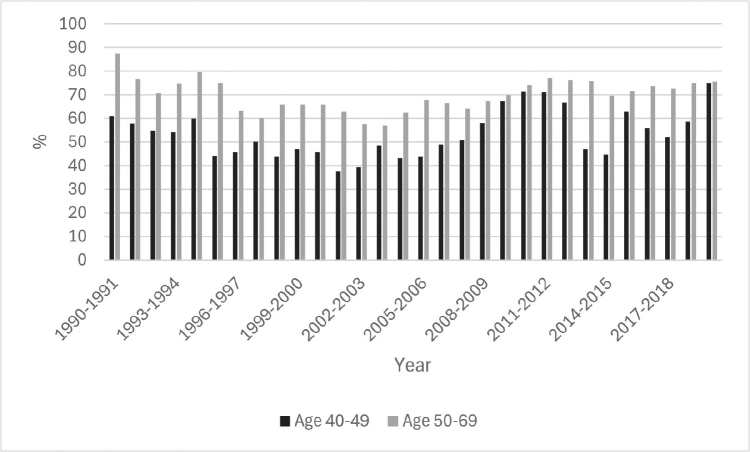
Episode sensitivity for screen-detected breast cancers in women aged 40–49 years and 50–69 years.

Figure 4 shows episode sensitivity for the age groups 40–49 and 50–69 years during the study period. Overall, episode sensitivity for women aged 40–49 years was lower when compared with women aged 50–69 years, or 54.8% versus 70.5%. Sensitivity seems to improve for younger women around 2010. Women aged 45–49 years also had higher episode sensitivity when compared with women aged 40–44 years, or 58.9% versus 49.0%.

## Discussion and conclusion

In this nationwide study spanning 30 years, both age groups (ages 40–49 and 50–69 years) met acceptable benchmarks on most breast cancer screening quality indicators, although screening attendance remained low among both age groups throughout the study period. We observed that women aged 40–49 years had a higher recall rate and lower values for PPV-1, breast cancer detection rate, interval cancer rate, and episode sensitivity compared to women aged 50–69 years. Furthermore, when the subgroups of women aged 40–49 years were analysed, the same trends were observed for the youngest group (age 40-44 years, although this group had the lowest PPV-1, breast cancer detection rate and episode sensitivity. Notably, among screen-detected women, those aged 40–49 years had a higher proportion of tumours larger than 20 mm, higher proportion of lymph node involvement, and higher proportion of HER2 positivity compared to women aged 50–69 years, a pattern not observed in clinically detected breast cancers.

When screening attendance, crucial for the effectiveness of screening programs, was compared with Denmark, Finland and Norway, Iceland had the lowest rate, with around 60% attendance during the study period (approximately 2001–2020), while the other countries reported significantly higher rates ranging from 77% to 84% [18]. Acceptable level of screening attendance according to European guidelines is considered to be 70% and 75% for the desirable level [17]. This observed difference may be partly explained by the application of a participation fee and absence of a pre-booking system in Iceland, whereas most other Nordic countries offered free screening and had a pre-booking system, both factors that are considered essential for improving screening attendance [19]. In 2019, the ICS waived the attendance fee for women attending their first screening, which significantly improved the overall attendance that year (see Supplementary Figure 1). However, as breast cancer screening was recently made free of charge and a pre-booking system is set to be implemented, participation rates in Iceland are expected to improve in coming years. This is of importance as it has been shown that increased attendance improves detection rates [20]. Another significant improvement in Iceland’s breast cancer screening program came in 2021, when the upper age limit for screening was extended to 74 years due to the high incidence of breast cancer in this age group [21].

The recall rate of this study was found to be within acceptable range according to the European guidelines, except for prevalent recalls for women aged 45–49 years. The reason for the temporary increase in recalls around 2010 remains unclear but may be linked to the introduction of digital imaging in Iceland in 2008. Digital imaging, which offers higher image quality compared to film [22], could also help explain the rise in the rate of screen-detected cancers and higher episode sensitivity during the same period. Similar patterns in both recall rates and cancer detection rates were observed in Norway following the implementation of digital imaging [23]. Increased recall rate can enhance breast cancer detection rates [24] but in our data, the rate of false positive recalls rose in accordance with the recall rate during the study period (Supplementary Figure 2), a trend that may also discourage women from returning for future screening [25, 26]. However, the effect of random fluctuations in breast cancer incidence in Iceland cannot be ruled out. Because of the small size of the Icelandic population, the number of breast cancer diagnoses varies greatly from year to year [27].

Higher incidence of breast cancer with increasing age can also explain the higher rate of screen-detected cancers among the older women, but this rate can depend on the age of participants, prevalence of risk factors and incidence of breast cancer in the population being screened. The lowest acceptable rate of screen-detected cancers for women older than 50 years is generally considered to be 4.1 per 1000 for prevalent screens and 4.3 per 1000 for subsequent screens [28]. The minimum rate is considered lower for women aged 40-49 years, or 2.5 per 1000 screens [29], which was not reached properly until the arrival of digital imaging. Lower incidence and higher recall rates for younger women also explain low PPV-1 in this age group, but PPV-1 was ≥ 16% in Norway [23].

Higher incidence of breast cancer in women over 50 might also explain the higher rate of interval cancer observed in our results. It has also been suggested that women who have participated in repeated screenings are at greater risk of being diagnosed with interval cancer compared to women attending their first screening [30]. The reason for this is unclear but may be due to lower recall rates in this group. However, different definitions of interval cancer, such as whether in situ tumours, false-negative assessments, and lapsed attenders are included or excluded, as well as accuracy in record-keeping, makes it difficult to determine the ideal frequency of interval cancers [31, 32]. A detailed review of interval cancers revealed that in most studies, where women were invited to screening every 2 years, the frequency of interval cancers ranged from 8.4 to 21.3 per 10,000 screens [33], which aligns well with our results.

One of the main reasons women aged 40–49 years, particularly those aged 40–44 years, appear to deviate more from optimal screening standards may be the combination of higher breast density and lower breast cancer incidence in this age group when compared with older women. Because both dense breast tissue and breast tumours appear white on a mammogram, distinguishing between the two can be challenging [34, 35]. This could complicate the diagnostic process and may also help explain the higher recall rates and lower episode sensitivity among younger women, due to the uncertainty in interpreting their mammograms, especially for the youngest age group, as breast cancer arising in a younger host is characterised by a more aggressive phenotype [36]. Older women might perform better as breast density gradually declines around the time of menopause, with continued reductions observed throughout the postmenopausal period [37, 38].

The masking effect of breast density in younger women, which can allow faster growing tumours to remain undetected for longer [39], may also partly explain the observed differences in tumour size and lymph node positivity between age groups among screen-detceted tumours. Studies have shown that shorter screening intervals in women aged 40–49 years might reduce mortality [30, 40–43] but would not necessarily be cost-effective [44]. Another reason may be because women who attend regular screening tend to have a higher underlying risk of breast cancer, possibly due to greater awareness stemming from family history or higher socioeconomic status [45–47]. Indeed, the prevalence of hereditary breast cancer is high among young cases. For example, 21% of women in Iceland diagnosed before the age of 40 have the Icelandic *BRCA2* founder mutation [48] and the prevalence of the mutation decreases rapidly with increasing age at diagnosis. A higher prevalence of hereditary cancer could therefore, to some extent, explain the less favourable tumour characteristics observed among women aged 40–49 when compared with women aged 50–69. A personalised approach would perhaps be the best way to determine age at first screening invitation and interval between invitations, and could be based on family history, breast density and polygenic risk scores, for example.

It is noteworthy that the categorical difference in tumour size and lymph node was not observed between the age groups among women with clinically diagnosed breast cancer, despite those women having a significantly higher proportion of tumours larger than 20 mm and a greater overall diameter across all age groups. Women who present with symptoms of breast cancer may have a more similar stage at diagnosis across age groups compared to those with screen-detected tumours found in symptom-free women.

Interestingly, no difference was observed between the age groups for invasive screen-detected cancers under 10 mm and all age groups were within limits of the European guidelines. This suggests that screening is equally sensitive at detecting small invasive cancers in both age groups. Nevertheless, younger women had a higher proportion of in situ breast cancer, suggesting some overdiagnoses for this group. Although in situ breast cancer is not considered life threatening and can remain indolent for quite some time, the proportion that will progress to being invasive and possibly life-threatening is currently unknown [49–51].

The main strengths of this study are the unique nationwide population data spanning 30 years, which is of particular importance as studies on quality of breast screening for women aged 40–49 years are limited. Another strength is the robust information from the Icelandic Cancer Registry, which is almost 100% complete for the entire population [52]. Also, due to Iceland’s unique personal identification number, used for all contact with the health care service and death register, as well as high-quality health registers, the follow-up of participants was virtually complete. However, the study also has some limitations. The use of information from Statistics Iceland, used to calculate screening attendance rate, might not represent the exact numbers invited for screening, resulting in either higher or lower attendance outcome. However, this difference is likely to be minimal and non-differential across age groups and thus would not substantially affect our main results. Another potential source of non-differential misclassification stems from challenges in classifying breast cancer cases as screen-detected or interval, particularly due to incomplete information for some women, especially those under clinical surveillance. This non-differential misclassification could bias results toward the null, potentially underestimating differences between screen-detected and interval cancer. However, some interval cancers, particularly among younger women with dense breast tissue might have been present but not detected at the time of the screening. This may have resulted in differential misclassification of quality indicators and tumour characteristics between the age groups. It should also be noted that Iceland has a small population, and the number of cancer cases each year, particularly among younger women, is relatively low. As a result, fluctuations in frequency may occur between years and should be interpreted with caution. Finally, the absence of breast density data limits our ability to interpret lower screening performance observed in younger women.

In conclusion, most quality indicators met acceptable thresholds among women who participated in the Icelandic population-based Breast Cancer Screening Program for 30 years. There were no apparent changes with time except for a slight increase in the detection rate for breast cancer. Screening attendance remained consistently low during the period and must be increased. However, lower performance across multiple indicators was observed for women aged 40–49 years, particularly those aged 40–44, compared with women aged 50–69 years. This disparity may be partly attributed to unmodifiable factors, such as lower incidence of breast cancer and higher breast density observed in the younger age group, making this a more difficult age to screen. Nevertheless, screening appears to detect small invasive cancers equally in both age groups and the presence of advanced tumours among screened women aged 40–49 years indicates the importance of early detection for improving prognosis.

## Supplementary Material





## Data Availability

Data cannot be made available due to legal restrictions; part of the data in this study were obtained from the national Icelandic Cancer Registry, where national data protection laws applies and forbids data from being publicly available.
