# Supplementary material for: Thirty years of population-based breast cancer screening in Iceland: a comparison of quality indicators and tumour characteristics between women aged 40–49 and 50–69 years
Source: Acta Oncol. 2025 Aug 14;64:44090. doi: 10.2340/1651-226X.2025.44090 (PMC12372527; doi:10.2340/1651-226X.2025.44090)
Supplement: Supplementary file 1 [file AO-64-44090-s1.pdf]

Supplementary material has been published as submitted. It has not been copyedited, or typeset by Acta Oncologica

Supplementary table 1a. Population-based breast cancer screening in Iceland, 1990-2020:
– quality indicators among women aged 45-49 and 40-44 years

Quality indicators	***Subgroups of age 40-49***		p-value
	**Age 45-49**	**Age 40-44**	
**Attendance, no, %**	94792 (60.2)	88904 (60.4)	0.484
Prevalent screen	46175 (49.3)	8042 (9.0)	< 0.001
Subsequent screens	48077 (50.7)	80862 (91.0)	< 0.001
**Age at screening, mean (SD)**	47,0 (1.4)	41.9 (1.5)	
**Recall rate, no. (%)**			
All screens	4222 (4.7)	4771 (5.0)	0.007
Prevalent screen	640 (8.0)	2875 (6.2)	< 0.001
Subsequent screens	3582 (4.4)	1896 (3.9)	< 0.001
**PPV-1, no. (%)**			
All screens	237 (5.6)	138 (2.9)	< 0.001
Prevalent screen	47 (7.3)	76 (2.6)	< 0.001
Subsequent screens	190 (5.3)	62 (3.3)	0.001
**Rate of screen detected-cancer, per 1000**			
All screens	2.8	1.5	< 0.001
Prevalent screen	6.1	1.7	< 0.001
Subsequent screens	2.5	1.4	< 0.001
**Rate of interval cancer, per 1000**			
All screens	2.0	1.6	0.089
Prevalent screen	2.4	1.6	0.100
Subsequent screens	1.9	1.6	0.282

Supplementary table 1b. Tumour characteristics among women aged 40–44 and 40–49 with screen-detected, interval, and clinically diagnosed invasive breast cancers, 1990–2020

Tumour characteristics	Screen-detected breast cancers			Interval breast cancers			Clinically diagnosed breast cancers		
	Age 45-49	***Age 40-44***	**p-value**	***Age 45-49***	***Age 40-44***	**p-value**	***Age 45-49***	***Age 40-44***	**p-value**
**All tumours, no**	248	146		173	152		192	166	
**Type (%)**									
In situ	47 (19.0)	29 (19.9)	0.825	11 (6.4)	17 (11.2)	0.122	5 (2.6)	10 (6.0)	0.080
Invasive**	201 (81.0)	117 (80.1)		162 (93.6)	135 (88.8)		187 (97.4)	156 (94.0)	
**Tumour diameter, mm**									
Median	15.0	15.5	0.675	18.0	20.0	0.675	20.0	21.0	0.512
Mean	18.2	19.1	0.593	23.1	22.5	0.814	27.9	26.3	0.722
All **<10 (%)**	56 (28.0)	34 (30.4)	0.699	27 (17.3)	19 (15.1)	0.615	26 (15.0)	19 (13.4)	0.677
Prevalent screen	9 (24.3)	16 (26.2)	0.834						
Subsequent screens	47 (29.1)	18 (35.3)	0.410						
All **10 ≥, <21, n (%)**	86 (43.4)	42 (37.5)	0.308	64 (41.0)	49 (38.9)	0.716	63 (36.4)	50 (35.2)	0.824
Prevalent screen	15 (40.5)	24 (39.3)	0.907						
Subsequent screens	71 (44.1)	18 (35.3)	0.267						
All **>20, n (%)**	56 (28.3)	36 (32.1)	0.475	65 (41.7)	58 (46.0)	0.462	84 (48.6)	73 (51.4)	0.614
Prevalent screens	13 (35.1)	21 (34.4)	0.943						
Subsequent screen	43 (26.7)	15 (29.4)	0.706						
**Positive node status, n (%)**									
All	77 (39.1)	50 (45.0)	0.308	84 (53.8)	68 (54.8)	0.869	81 (46.8)	62 (45.3)	0.784
Prevalent screen	16 (43.2)	29 (47.5)	0.060						
Subsequent screens	61 (38.1)	21 (42.0)	0.010						
**ER, n (%)**									
Positive	165 (85.5)	85 (79.4)	0.531	113 (72.9)	87 (68.5)	0.574	126 (71.6)	103 (72.5)	0.846
**HER2, n (%) *****									
Positive	8 (22.6)	8 (11.3)	0.474	20 (21.3)	8 (11.0)	0.077	19 (3.7)	14 (2.7)	0.273
