# Supplementary material for: Thirty years of population-based breast cancer screening in Iceland: a comparison of quality indicators and tumour characteristics between women aged 40–49 and 50–69 years
Source: Acta Oncol. 2025 Aug 14;64:44090. doi: 10.2340/1651-226X.2025.44090 (PMC12372527; doi:10.2340/1651-226X.2025.44090)
Supplement: Supplementary file 2 [file AO-64-44090-s2.pdf]

Supplementary material has been published as submitted. It has not been copyedited, or typeset by Acta Oncologica

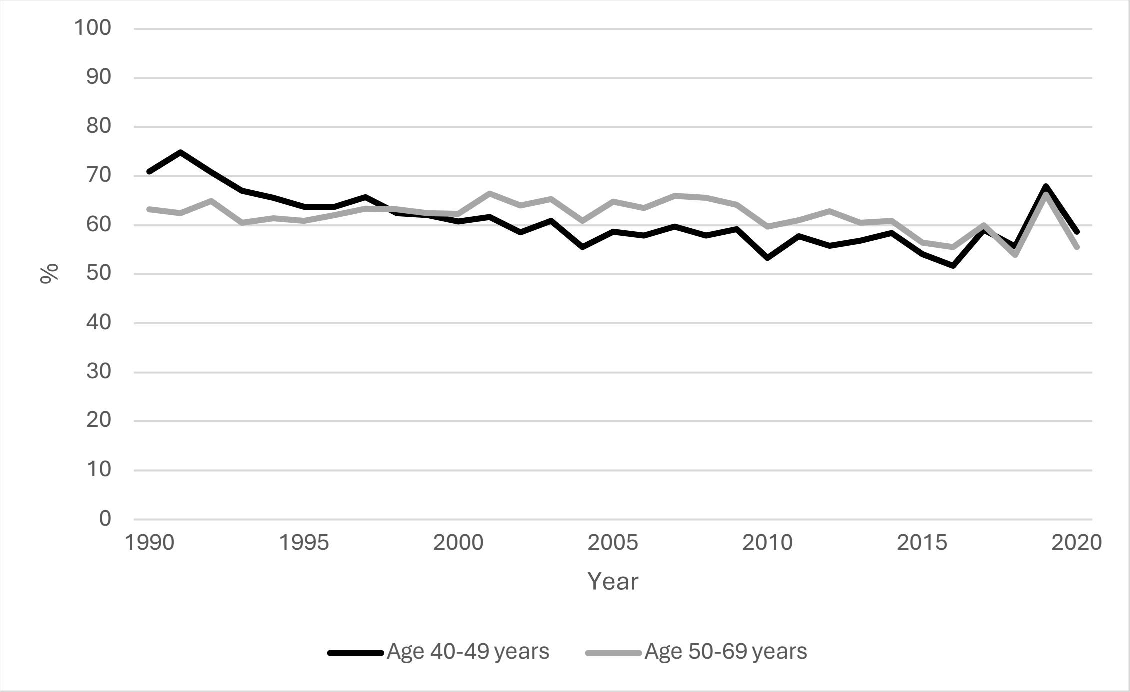

Supplementary figure 1. The breast cancer screening attendance rate among women aged 40-49 and 50-69 years during the years 1990-2020.

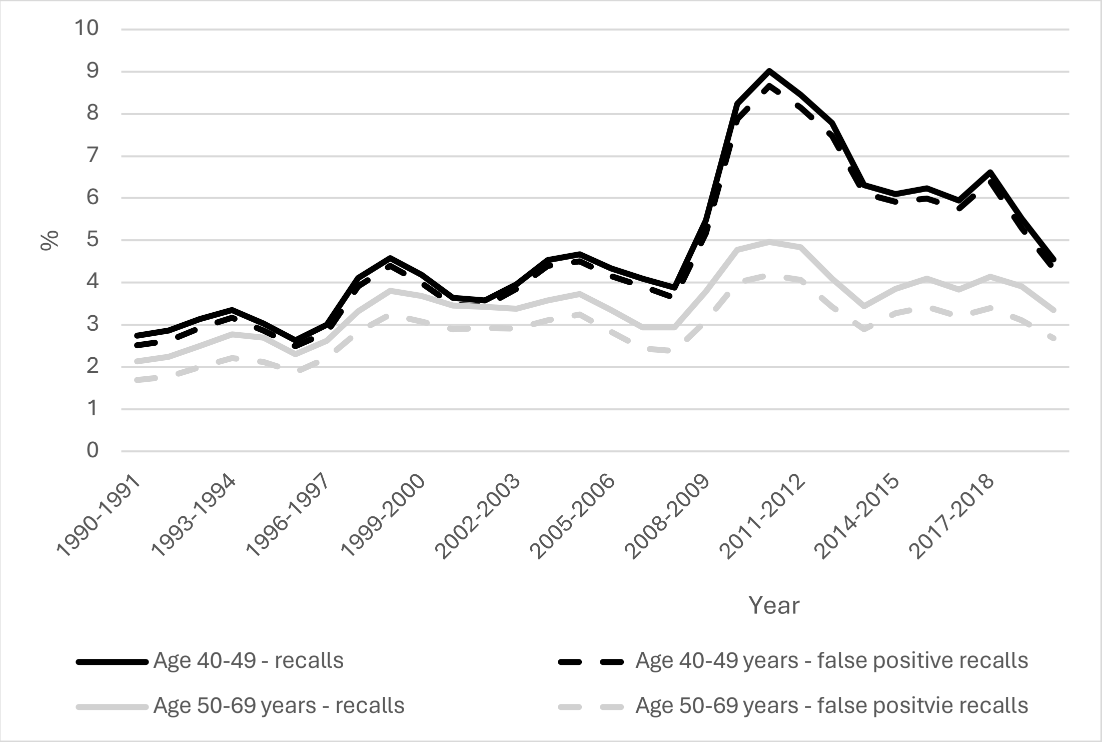

Supplementary figure 2. Rate of recalls and false positive recalls among women aged 40-49 and 50-69 years during the years 1990-2020.
